# The aminoadamantane derivative 3F2 modulates monocyte and NK cell activation and restores immune mediators altered by Monkeypox virus stimuli

**DOI:** 10.1007/s00011-026-02387-3

**Published:** 2026-10-17

**Authors:** Thaís de Fátima da Silva Moraes, Geovane Marques-Ferreira, Ágata Lopes-Ribeiro, Erik Vinicius de Sousa Reis, Gabriela Lorrany Aparecida Azevedo, Victor de Melo Rocha, Marília Bueno da Silva Menegatto, Felipe Alves Clarindo, Caio Wilker-Teixeira, Laura Cardoso Corrêa-Dias, Nathália Evelyn Morais Costa, Luan Ramalho Pinheiro, Adriano de Paula Sabino, Erna Geessien Kroon, Ângelo de Fátima, Jordana Grazziela Alves Coelho-dos-Reis

**Affiliations:** 1 ProsPERA Group, Grupo de Prospecção e Pesquisas em Resposta Imune e Antivirais, Universidade Federal de Minas Gerais, Avenida Antônio Carlos, 6627, Pampulha, Belo Horizonte, MG Brazil; 2 Departamento de Microbiologia, Instituto de Ciências Biológicas, Universidade Federal de Minas Gerais, Avenida Antônio Carlos, 6627, Pampulha, Belo Horizonte, MG Minas Gerais–31270-901, Brazil; 3 Departamento de Química, Instituto de Ciências Exatas, Universidade Federal de Minas Gerais, Avenida Antônio Carlos, 6627, Pampulha, Belo Horizonte, MG Brazil; 4 Laboratório de Hematologia Clínica, Experimental e Molecular, Departamento de Análises Clínicas e Toxicológicas, Faculdade de Farmácia, Universidade Federal de Minas Gerais, Avenida Antônio Carlos, 6627, Pampulha, Belo Horizonte, MG Brazil

**Keywords:** Mpox, MPV, Immunomodulators, Viral immunity, Aminoadamantane

## Abstract

The monkeypox virus (MPV) is known to employ multiple immune evasion strategies that compromise antiviral defenses and disrupt immune homeostasis. Here, we characterized the immune responses induced by stimulation with inactivated MPV and evaluated the ability of aminoadamantane (AMA) and its derivative, 3F2, to restore immune function. Peripheral blood mononuclear cells that were stimulated with MPV exhibited altered activation in a subset of monocytes, along with suppressed activation of natural killer (NK) and natural killer T (NKT) cells. MPV stimulation also led to significant changes in the levels of immunosoluble mediators, including reductions in chemokines and cytokines that are crucial for antiviral defense and immune regulation. Treatment with 3F2 proved to be more effective than AMA, resulting in significantly higher levels of CCL2, CXCL8, IFN-γ, IL-12(p40), IL-15, IL-5, and essential growth factors such as VEGF and NGF-β. Furthermore, 3F2 treatment partially reverses monocyte activation and re-establishes NK and NKT cell function. The current results indicate that 3F2 can control inflammation while preserving antiviral responsiveness. These findings demonstrate that inactivated MPV stimuli profoundly modulates innate immune activation and identify 3F2 as a promising immunomodulatory candidate capable of partially restoring immune responses altered by viral particle stimulation.

**Supplementary Information:**

The online version contains supplementary material available at https://doi.org/10.1007/s00011-026-02387-3.

## Introduction

Since 2022, the mpox (formerly known as monkeypox disease) outbreak has spread quickly. Until December 2025, more than 175,415 cases and 467 deaths have been reported across 142 countries, including at least 103 nations with no previous history of mpox [1–2]. Mpox is caused by the *Orthopoxvirus monkeypo*x (MPV), a member of the genus *Orthopoxvirus* [3].

Poxviruses use multiple strategies to evade the host’s immune responses, including the production of viral proteins that interfere with host signaling pathways involved in viral recognition, apoptosis, and immune regulation [4]. In MPV infection, innate immune cells serve as the first line of defense. Monocytes and granulocytes are among the early targets of infection and contribute to viral dissemination and the evasion of neutralizing antibodies [5]. Natural killer (NK) cells play a central role in antiviral defense against MPV through cytotoxic activity and cytokine production. NK cells migrate to sites of infection via chemokine receptor signaling, thereby coordinating both innate and adaptive immune responses [4, 6].

Other immune evasion strategies of poxviruses are the secretion of viral proteins that mimic host cytokines or cytokine receptors, which act as decoy molecules that suppress cytokine-mediated signaling. These strategies are further complemented by inhibiting NK cell and cytotoxic T cell activity, often by disrupting antigen presentation pathways [5, 7]. MPV infection can also induce severe immunopathological responses, including excessive inflammation and damage to immune organs such as the thymus, lymph nodes, and spleen [5].

The United States Food and Drug Administration (FDA) approved the JYNNEOS vaccine against MPV in 2019, however, it does not confer complete protection [8]. Regarding mpox therapies, to date, no specific antiviral therapy has been fully approved, and clinical management primarily relies on symptomatic treatment and control of complications [9]. Although tecovirimat, cidofovir, and brincidofovir, originally authorized for smallpox, have been considered for mpox treatment, recent Phase II clinical trials demonstrated a lack of efficacy of tecovirimat in individuals infected with MPV Clade I [4, 10–15].

In this context, a promising therapeutic strategy is the use of antiviral agents with immunomodulatory properties, which restore antiviral immunity, modulate inflammation, and improve clinical outcomes by selectively regulating host immune signaling pathways and key immune cell populations [16–18].

Amantadine, also known as aminoadamantane (AMA), was the first antiviral approved for treating Influenza A. In addition to antiviral activity, it has immunomodulatory properties, particularly in regulating inflammatory responses. However, its relevance during viral infections remains poorly understood [19–22]. A previous study demonstrated the robust antiviral activity of an AMA derivative, 3F2, against MPV infection, underscoring its potential as a therapeutic candidate [23]. The present study explores the immunomodulatory properties of AMA and 3F2 on MPV stimulation in vitro, focusing on their ability to restore immune response balance disrupted by viral challenge.

## Methods

### Chemical compounds

The AMA derivative (3F2) was synthesized and characterized as previously described [22] de Almeida Marques et al., 2024). AMA (Sigma-Aldrich, Merck, USA) and 3F2 were dissolved in sterile dimethyl sulfoxide (DMSO; Sigma-Aldrich, USA) and stored at -20 °C until use.

### Monkeypox virus (MPV)

MPV (BR0001/2022-GenBank: ON751962.1) was replicated and titrated in a biosafety level 3 (BSL-3) laboratory at the Instituto de Ciências Biológicas, Universidade Federal de Minas Gerais. Briefly, BSC-40 cells were infected at a multiplicity of infection (MOI) of 0.01 for 48 h, and viral titers were determined using the Dulbecco plaque assay, as previously described [23–25]. For use in biological assays, MPV was inactivated by treatment with 0.5% paraformaldehyde (Neon Comercial Ltda., Brazil), followed by UV exposure for 1 h. This research was registered on the SISGEN platform (access A43330C).

### Isolation of peripheral blood mononuclear cells (PBMC)

Peripheral blood mononuclear cells (PBMCs) were isolated from healthy donors using a Ficoll-Paque gradient as previously described [26], with some modifications. Whole blood from healthy donors collected in heparin tubes was centrifuged at 1960 × g for 10 min at room temperature (RT) to remove plasma, followed by the addition of the same volume of incomplete RPMI 1640 medium (Gibco, Thermo Fisher Scientific, USA). After homogenization, the diluted blood was slowly layered onto a Ficoll solution (Ficoll-Paque Plus, density 1.077 g/mL; Cytiva, USA) using a serological pipette. Following, a centrifugation at 490 × g for 40 min at room temperature was performed, and a well-defined PBMC ring was observed at the Ficoll interface. The buffy coat was removed using a Pasteur pipette and transferred to a 50 mL tube. Next, two washes were performed by adding 20 mL of incomplete RPMI 1640 medium and centrifuging the tubes for 7 min at 706 × g at 4 °C. The cell pellet was resuspended in 1 mL of RPMI 1640 medium supplemented with 10% SFB.

### In vitro stimulation of PBMCs with MPV and treatment with AMA and 3F2

PBMCs were cultured in duplicate in 96-well plates (5 × 10^5^ cells/well) and maintained in RPMI 1640 medium (Gibco, USA) supplemented with 10% FBS, streptomycin (100 µg/mL; Sigma-Aldrich, USA), potassium penicillin (100 U/mL; Sigma-Aldrich, USA), and amphotericin B (2.5 µg/mL; Sigma-Aldrich, USA). PBMC cultures were stimulated with inactivated MPV at 0.01 viral particle equivalents per cell (VPE) in the presence of AMA or 3F2 (100 µM). For each donor, cell control (PFA added), virus control, DMSO control, and treatment with AMA and 3F2 were performed. The cultures were incubated for 12 h in a humidified incubator at 5% CO_2_ and 37 °C. After the incubation period, the plates were centrifuged at 500 × g for 10 min at 18 °C. Culture supernatants were collected and stored for subsequent analysis of soluble factors (Sect. 2.6), and the cells were harvested and stained for immunophenotyping by flow cytometry (Sect. 2.5) [27].

### Immunophenotyping assay

Immunophenotyping of immune cell subpopulations was performed using a panel of fluorochrome-conjugated monoclonal antibodies (Supplementary Table S1). Briefly, cells were resuspended in 20 µL of LIVE/DEAD™ Fixable Aqua Dead Cell Stain (Thermo Fisher Scientific) and incubated for 5 min at RT. Cells were incubated with the antibodies for 30 min at RT, except for the unstained control. Next, 150 µL of 1× PBS was added, and the cells were washed at 1190 ×g for 10 min at 18 °C. The cells were resuspended in PBS for subsequent acquisition in an LRS Fortessa flow cytometer (BD Biosciences, San Jose, CA, USA). The FlowJo V10.8.1 software (BD Biosciences, San Jose, CA, USA) was used for data analysis using distinct gating strategies.

### Quantification of immunosoluble mediators

The analysis of immunological biomarkers was performed using the supernatant obtained from the in vitro system described in Sect. 2.4. The PBMC culture samples were centrifuged at 800xg for 10 min at RT and diluted 1:2 for further analysis. The Luminex Bio-Plex Pro™ human cytokines platform (Bio-Rad, #12007283) was utilized to quantify systemic immunosoluble mediators, enabling the investigation of 48 analytes, including chemokines (CXCL1, CCL2, CCL3, CCL4, CCL5, CCL7, CXCL8, CXCL9, CXCL10, CCL11, CXCL12 and CXCL27), pro-inflammatory cytokines (IFN-a2, IL-1α, IL-1β, IL-6, IL-12(p70), IL-12(p40), TNF-α, TNF-β, TRAIL, IFN-γ, IL 15, IL-16, IL-17a and IL-18), regulatory cytokines (IL-1Ra, IL-2Ra, IL-4, IL-5, IL-9, IL-10, IL-13 and MIF), and growth factors (HGF, β-NGF, FGF-basic, PDGF, VEGF, SCF, SCGF-basic, IL-3, G-CSF, GM-CSF, LIF, IL-2 and IL-7). The samples were tested according to the manufacturer’s instructions, and the measurements were performed on a BioPlex 200 Instrument (Bio-Rad). The results were expressed in pg/mL concentration for all immunosoluble mediators in the culture supernatants of the different treatments.

### Statistical analysis

Statistical analyses were performed using GraphPad Prism (version 9.0). Differences between the two groups were determined using the Wilcoxon non-parametric test with Dun’s post-test. A *p*-value ≤ 0.05 was considered statistically significant.

## Results

### 3F2 derivative modulates MPV-induced activation of monocytic subsets by decreasing HLA-DR expression on the surface of patrolling monocytes

**Fig. 1:**
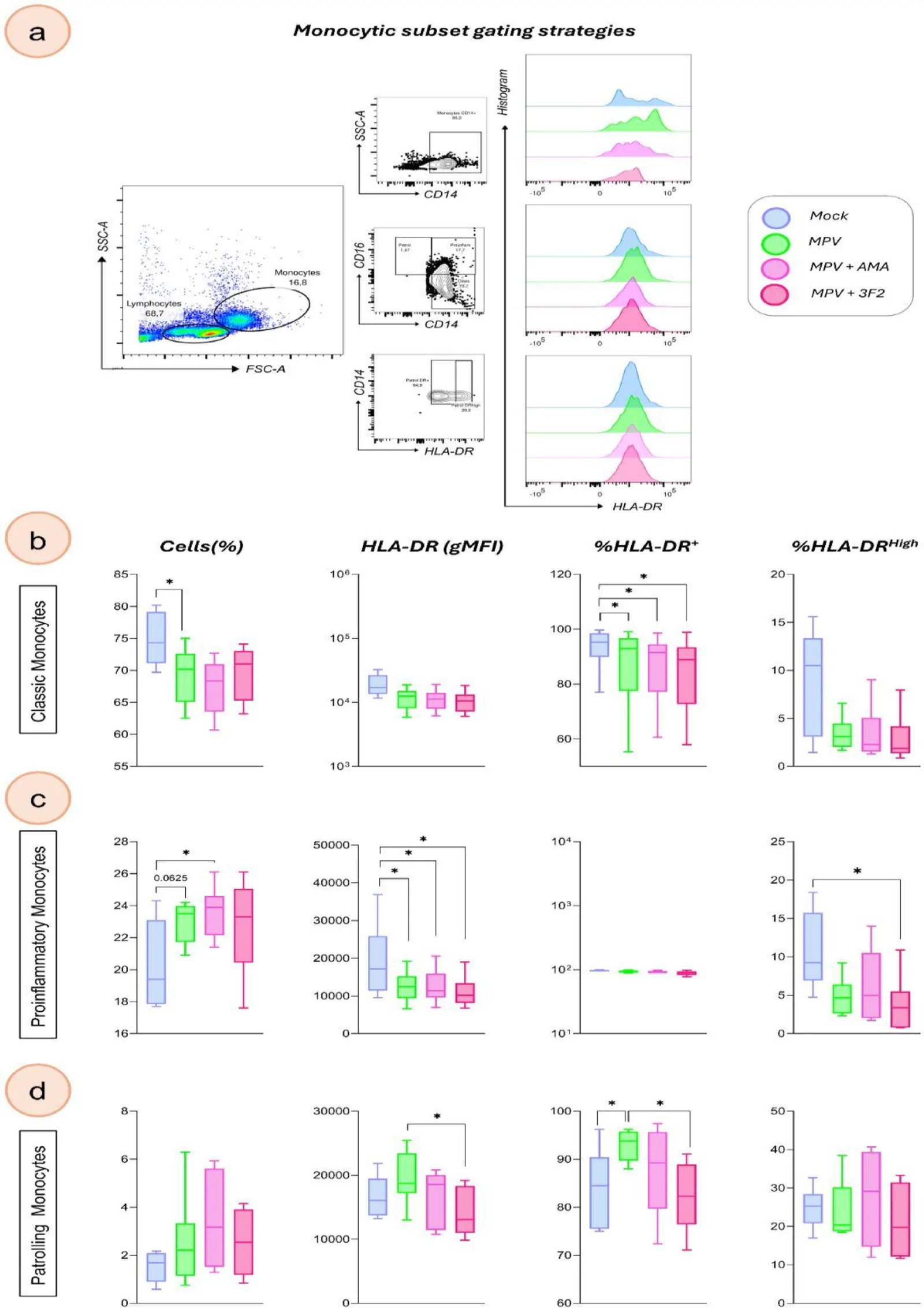
3F2 derivative modulates MPV-induced activation of monocytic subsets. **a** Flow cytometry gating strategy for the identification of CD14^+^ monocyte subsets. Sequential gating included: the monocyte population (SSC-A vs. FSC-A), CD14^+^ monocytes, and subsequent subdivision into classical, pro-inflammatory, and patrolling subsets. Representative histograms illustrate HLA-DR expression across experimental conditions. The frequency (%) and geometric mean fluorescence intensity (gMFI) of HLA-DR expression were analyzed in **b** classical, **c** pro-inflammatory, and **d** patrolling subsets. Data obtained from six independent donors (*n* = 6) are presented as box plots, showing minimum to maximum values. Statistical significance was determined using the Wilcoxon test, with *p* < 0.05 indicating significant differences

Figure 1 shows the results for the levels of Monocytic subsets, namely CD16^−^CD14^+^ (classic), CD16^+^CD14^+^ (pro-inflammatory), and CD16^+^CD14^low^ (patrolling) monocytes. MPV has decreased the levels of classic monocytes (Fig. 1b), while demonstrating a tendency (*p* = 0.0625) to induce an increase in pro-inflammatory monocytes (Fig. 1c). The levels of activation of these cells were evaluated by HLA-DR expression either in terms of percentage or geometric mean fluorescence intensity (gMFI). The data have demonstrated that MPV stimuli decreased the HLA-DR levels in classic and pro-inflammatory monocytes (% and gMFI, respectively), and a viral-induced activation was observed in patrolling monocytes. This subset activation was further modulated by 3F2. In the compound controls (Supplementary Table S1), the compounds modulate monocyte subset distribution and increased HLA-DR expression, indicating changes in the phenotypic state of monocytes.

### 3F2 derivative reconstitutes early and late steady-state activation markers reduced by MPV stimuli in NK subsets

**Fig. 2:**
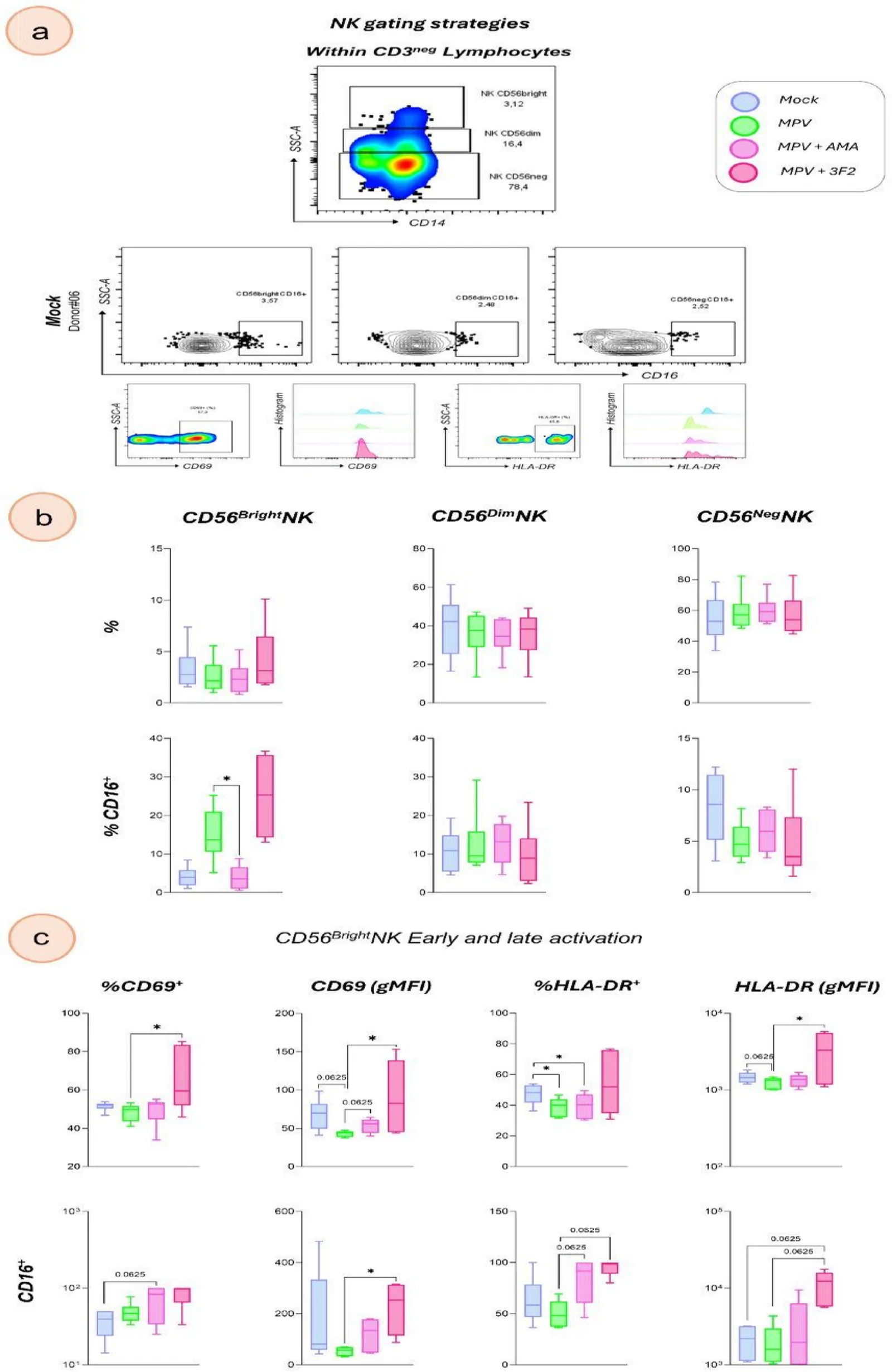
3F2 restores MPV-suppressed early and late NK cell activation. **a** Flow cytometry gating strategy for identification of NK cells (CD56^Bright/dim/neg^CD16^+^NK). **b** Frequency of CD56^Bright/dim/neg^CD16^+^NK cells across experimental groups. **c** Activation profile of CD56^Bright^CD16^+^NK subsets based on CD69 and HLA-DR expression, shown as frequency (%) and geometric mean fluorescence intensity (gMFI). Data obtained from six independent donors (*n* = 6) are presented in a box with minimum to maximum values. Statistical significance was determined using the Wilcoxon test, with *p < 0.05 indicating significant differences

Figure 2 illustrates the data for NK cells, including CD56^Bright/dim/neg^, CD16^+^NK, and subset activation profiles. The percentage of these cells (CD56^Bright/dim/neg^NK) remained unchanged by either MPV, AMA, or 3F2 treatments (Fig. 2b). However, MPV stimuli tended to increase the proportion of CD56^Bright^CD16^+^NK cells, a transitional subpopulation characterized by enhanced cytotoxic potential and increased responsiveness to inflammatory cues. Interestingly, AMA treatment restored CD56^Bright^CD16^+^NK cell levels relative to the MPV group, suggesting that AMA may mitigate the increase in this cell subset induced by MPV stimuli. In contrast, 3F2 treatment showed a tendency to promote a more pronounced increase in CD56^Bright^CD16^+^NK cells, suggesting a potential stimulatory effect on this population. Stimulation with MPV slightly reduced the CD56^Dim/neg^CD16^+^NK population, while AMA treatment partially restored this subset, showing a trend toward levels comparable to the mock condition, but with no statistical differences. The activation profiles (Fig. 2c) indicate that MPV stimuli was associated with a trend toward reduced CD69 and HLA-DR expression in CD56^Bright^ and CD16^+^CD56^Bright^ NK cells compared with the Mock group, although these differences were not statistically significant. Treatment with the 3F2 derivative significantly increased CD69 expression (frequency and gMFI) and HLA-DR expression (gMFI) in CD56^Bright^ NK cells compared with the MPV group. In CD16^+^CD56^Bright^ NK cells, 3F2 significantly increased CD69 surface expression (gMFI), whereas HLA-DR showed a trend toward increased expression (gMFI) and frequency (%) following treatment (*p* = 0.0625). These findings suggest that 3F2 modulates the activation phenotype of CD56^Bright^ NK-cell subsets during MPV stimulation. Although the compound controls (Supplementary Table S2) altered the distribution of CD56^Bright/dim/neg^CD16^+^NK cells, these changes were not accompanied by comparable alterations in the activation markers evaluated. Therefore, the enhanced expression of activation markers observed following 3F2 treatment in MPV-stimulated cells may reflect a context-dependent effect associated with MPV stimulation. Finally, MPV stimulation induced only limited changes in the activation profile of CD56^Dim/neg^CD16^+^ NK-cell subsets, and neither AMA nor 3F2 significantly altered these responses (Supplementary Fig. S1).

### AMA and 3F2 derivatives partially restore MPV-induced modulation of NKT cell activation markers

**Fig. 3:**
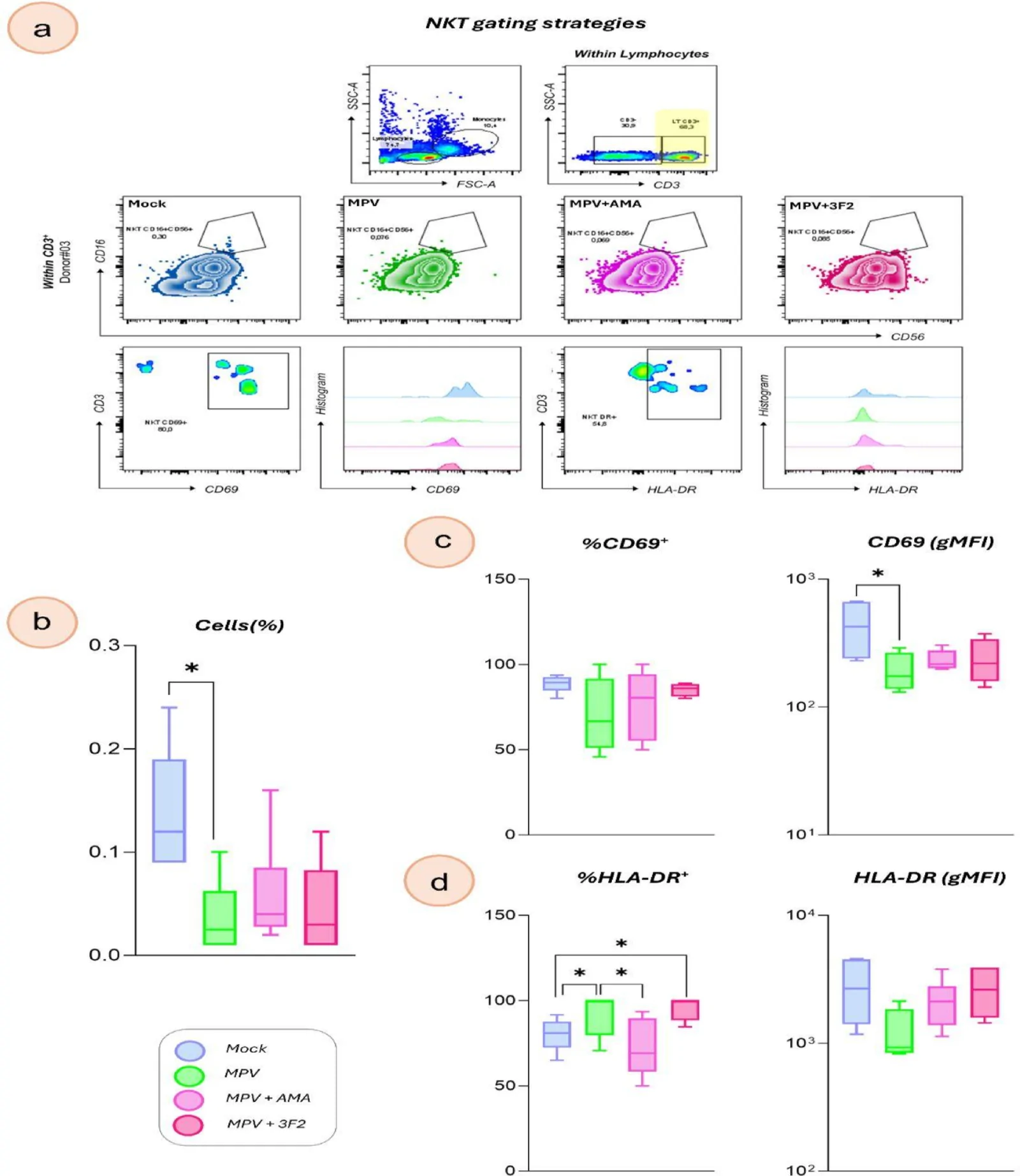
3F2 restores MPV-induced changes in NKT cell activation markers, partially **a** Flow cytometry gating strategy for identification of NKT cells (CD3^+^CD16^+^CD56^+^). **b** Frequency of CD16^+^CD56^+^NKT cells across experimental groups. Activation profile of NKT subsets based on **c** CD69 and **d** HLA-DR expression, shown as frequency (percentage) and geometric mean fluorescence intensity (gMFI). Data obtained from six independent donors (*n* = 6) are presented in a box with minimum to maximum values. Statistical significance was determined using the Wilcoxon test, with *p < 0.05 indicating significant differences

Figure 3 shows the frequency and activation profile of NKT cells (CD3⁺CD56⁺) under different experimental conditions. MPV stimuli significantly reduced the proportion of CD16⁺CD56⁺ NKT cells (Fig. 3b) and CD69 expression (gMFI) (Fig. 3c), whereas HLA-DR expression (gMFI) showed a tendency to decrease following MPV stimulation (Fig. 3d). Together, these findings are consistent with a reduction in the activation profile of NKT cells. Treatment with AMA and its derivative, 3F2, partially reversed this downregulation, restoring CD69 expression to near-baseline levels (Fig. 3c). Moreover, MPV stimulation increased the frequency of HLA-DR⁺ NKT cells, an effect that AMA attenuated (Fig. 3d). The compound controls (Supplementary Table S2) showed no major changes in NKT-cell frequency or activation profile compared with the Mock condition. These findings suggest that the 3F2 derivative modulates MPV-induced NKT activation; however, the restoration of activation markers did not reach statistical significance, indicating a limited immunomodulatory effect under these experimental conditions.

### Neither AMA nor 3F2 treatments restore the suppressed activation profile of lymphocytes following MPV stimulation

Finally, B and T cell subsets were analyzed for both frequencies, early (CD69) and late (HLA-DR) activation expression (Supplementary Fig. S2). It was observed that the MPV stimulation resulted in a slight increase in the frequency of total lymphocytes. Within this population, a subtle rise in B cells (CD3^−^CD19^+^) was detected, without evidence of cell activation, as indicated by the unaltered expression of HLA-DR and CD69 in MPV in comparison to Mock. Treatment with AMA or 3F2 did not induce significant changes in this profile, which remained similar to that observed in the group stimulated with MPV. Similarly, CD4⁺ and CD8⁺ T cell subsets (CD3^+^CD4^+^ and CD3^+^CD8^+^) displayed stable frequencies following MPV stimulation, although a reduction in their activation status was evident. Once again, treatment with AMA or 3F2 failed to restore the activation profile observed in the control group. The compound controls (Supplementary Table S2) showed reduced frequencies of total lymphocytes and CD3⁻CD19⁺ B cells, together with increased expression of CD69 and HLA-DR gMFI in lymphocyte subsets, indicating that the compounds themselves induced some phenotypic changes in the lymphocyte compartment. However, these effects were not associated with restoration of the lymphocyte alterations induced by MPV stimulation.

### 3F2 restores innate immune mediator secretion following MPV stimulation

**Fig. 4:**
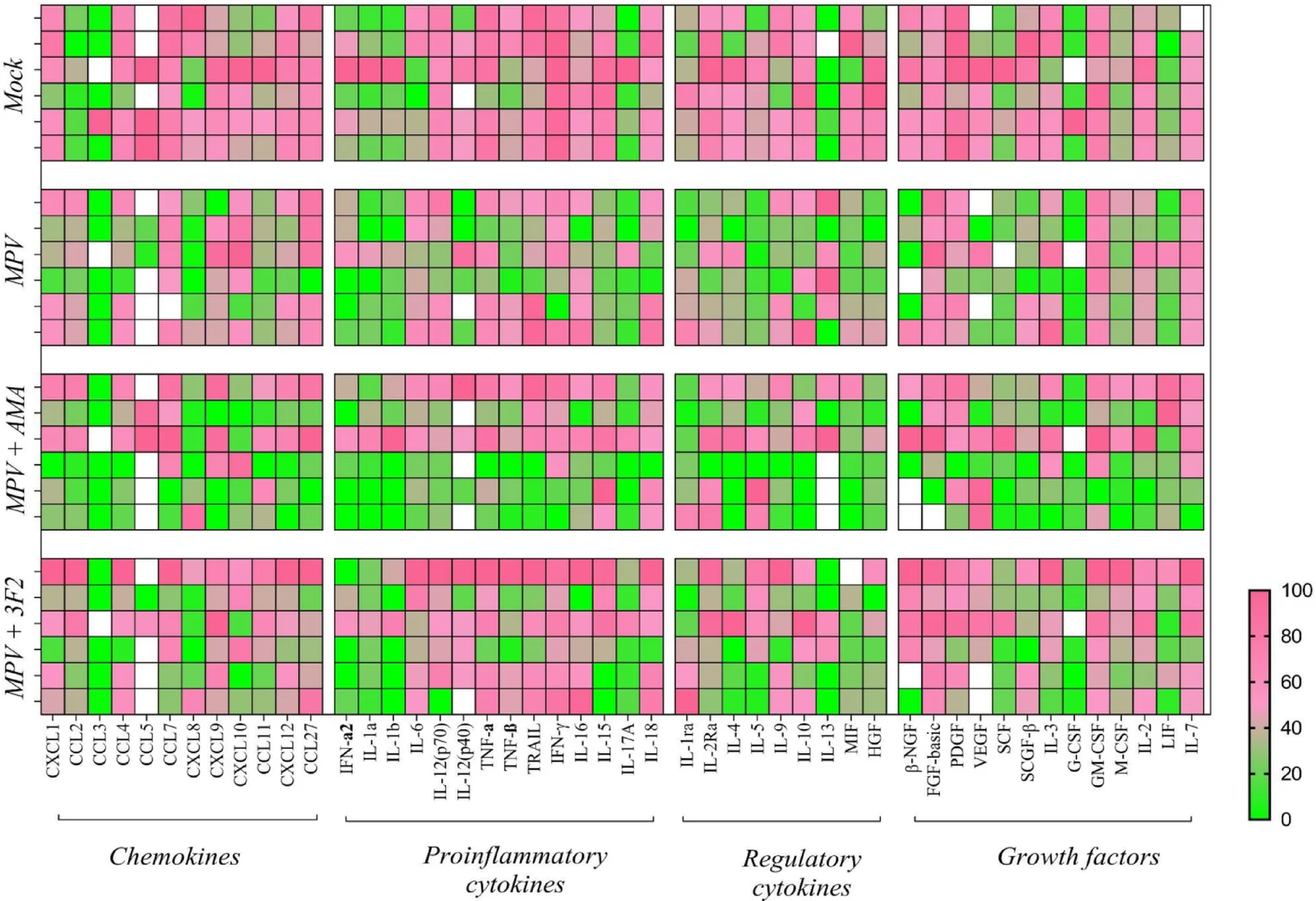
Differential modulation of soluble immune mediators by AMA and 3F2 derivative during MPV stimulation. Heatmaps show the modulation of soluble mediators grouped into functional categories, including chemokines, pro-inflammatory cytokines, regulatory cytokines, and growth factors, across the experimental conditions: Mock, MPV, MPV + AMA, and MPV + 3F2. Blank boxes indicate non-detectable or excluded values. Data represent individual normalized (with minimum to maximum values considered from 0 to 100) values obtained from six independent donors (*n* = 6)

To comprehensively characterize the immunomodulatory effects induced by MPV stimulation and subsequent treatment with AMA or its 3F2 derivative, the concentrations of chemokines, pro-inflammatory and regulatory cytokines, as well as growth factors, were quantified in PBMC culture supernatants using a 48-plex Luminex^®^ assay. Figure 4 illustrates the representative heatmaps showing the production profiles observed in untreated and unstimulated cells (mock), cells stimulated with MPV (MPV), and cultures stimulated with MPV treated with AMA or 3F2, respectively. Overall, MPV stimulation of PBMCs resulted in a broad reduction in the secretion of immune mediators across multiple functional categories, as reflected by the predominance of lower-intensity signals in the heatmap (Fig. 4). In contrast, treatment with the 3F2 derivative partially restored the secretion profile of innate immune mediators, indicating a reprogramming of the immune environment altered by MPV stimulation.

**Fig. 5:**
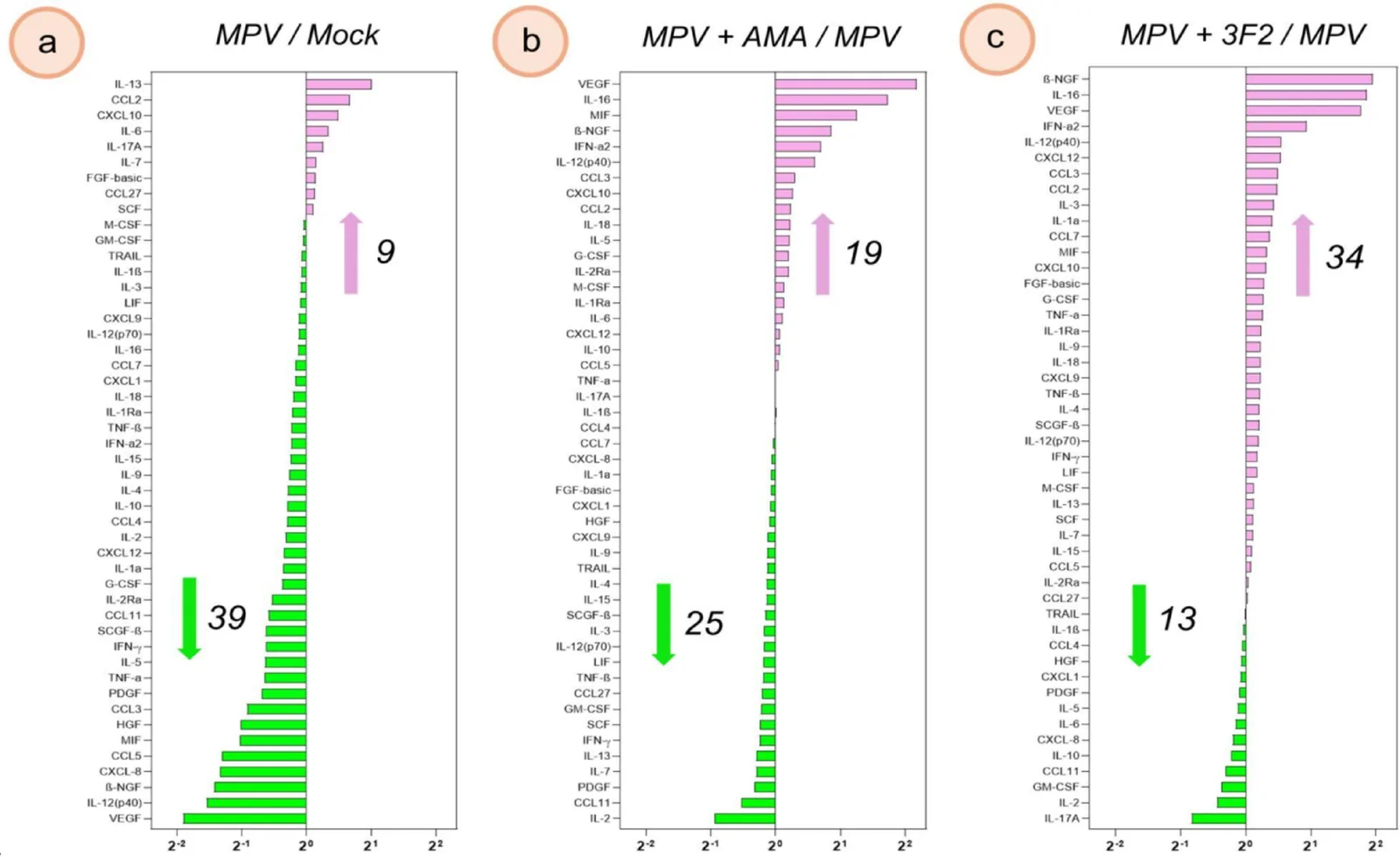
AMA and its 3F2 derivative modulate soluble immune mediators suppressed by MPV stimulation. Fold-change analysis of cytokine, chemokine, and growth factor production relative to the mock condition. Data obtained from PBMCs of six independent donors (*n* = 6) are presented as fold-change for **a** MPV/mock, **b** MPV + AMA/ MPV, and **c** MPV + 3F2/ MPV. Results are displayed as ordered bar plots illustrating the magnitude and direction of modulation of soluble immunological mediators following MPV stimulation and treatment with AMA or its 3F2 derivative

Consistent with an immunoevasive profile, the fold-change analysis revealed that MPV stimulation markedly suppressed the immune response, leading to decreased levels of 39 of the 48 evaluated mediators compared with mock cultures (Fig. 5a). In contrast, AMA treatment promoted a more balanced modulation of immune mediators, increasing 19 and reducing 25 mediators relative to MPV-stimulated cells (Fig. 5b). More prominently, treatment with the 3F2 derivative elicited a broader immunostimulatory effect, enhancing the expression of 34 mediators while reducing 13, suggesting a restoration of immune activity suppressed by MPV stimulation (Fig. 5c).

**Fig. 6:**
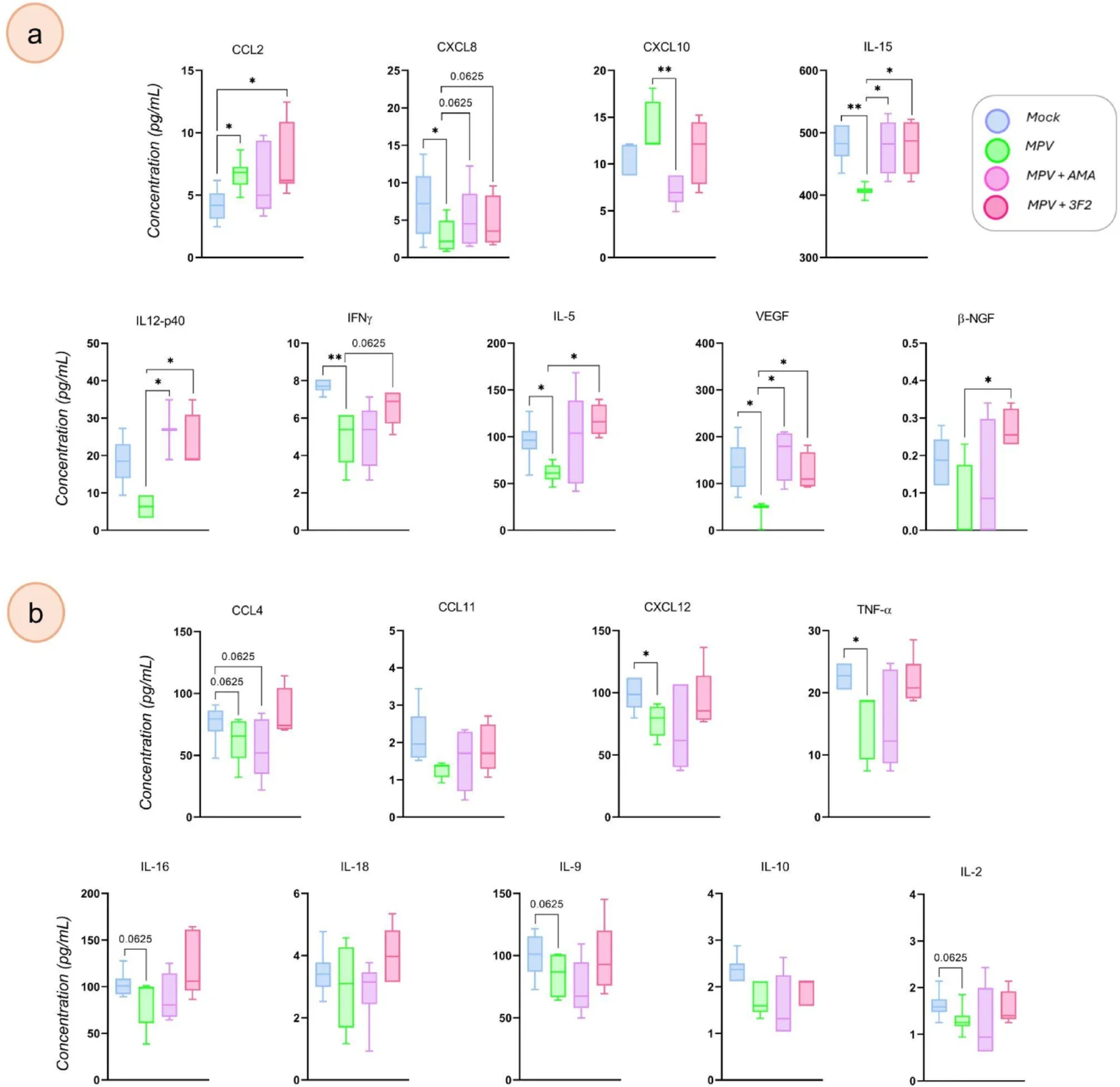
AMA and the 3F2 derivative modulate MPV-induced alterations in soluble immune mediators. **a** Representative graphs of proinflammatory cytokines, chemokines, regulatory mediators, and growth factors that showed statistically significant modulation upon MPV stimulation and/or treatment with AMA or the 3F2 derivative. **b** Profiles of immune mediators did not show statistical significance but displayed consistent trends of modulation across experimental conditions. Data represent the concentrations of soluble immune mediators measured in PBMC cultures from six independent donors (*n* = 6) under different experimental conditions (Mock, MPV, MPV + AMA, and MPV + 3F2). They are presented as box plots showing minimum to maximum values. Statistical significance was determined using the Wilcoxon test, with *p < 0.05 and **p < 0.01 considered significant

To further understand these effects, Fig. 6a presents immune mediators that were significantly modulated by MPV stimulation and subsequently affected by treatment with AMA or the 3F2 derivative, shown individually, highlighting distinct patterns of response across experimental conditions. Treatment with AMA, and more prominently with the 3F2 derivative, significantly modulated a subset of these mediators, with levels returning to values comparable to those observed under mock conditions, reinforcing the effect of immune restoration following MPV-induced suppression.

In addition to statistically significant changes, a broader set of immune mediators displayed consistent trends of modulation in response to treatment, although these did not reach statistical significance (Fig. 6b). The compound controls (Suplemmentary Table 2) also showed distinct patterns of mediator production, with selected inflammatory and regulatory mediators, increasing, the levels of IFN-γ, CXCL8, CXCL10, and IL-10. Together, these data indicate that AMA and the 3F2 derivative influence MPV-induced immune alterations, affecting both significantly regulated mediators and those showing coordinated directional trends.

## Discussion

Orthopoxviruses, including MPV, have strategies to evade innate immune recognition, allowing successful infection and viral persistence. For this, MPV employs various mechanisms to suppress immune activation and interrupt antiviral responses [7, 28]. Therefore, understanding the immune evasion mechanisms of MPV is essential for the development of effective antiviral therapies [5].

In this study, an in-depth investigation of the effect of MPV stimulation on PBMCs and whether AMA and its derivative 3F2 could modulate the immune changes induced by viral stimulation. The 12 h stimulation time point was selected to evaluate early innate immunity, as short-term stimulation (6–12 h) has been previously used to characterize early perturbations in innate immune genes and proteins and is sufficient to induce measurable immune responses to viral antigens [27–31]. In the present study, inactivated MPV preparation was employed to assess the capacity of preserved viral structural components and associated pathogen-associated molecular patterns (PAMPs) to activate innate immune pathways and induce measurable immune responses in PBMC cultures. Our findings support that viral structure proteins alone are able to induce innate immunity activation and can be, therefore, useful for mechanistic immunomodulatory studies [30, 32]. Importantly, the observed immunological changes were not associated with reduced cell viability, indicating that the effects observed primarily reflect immune modulation after stimulation with MPV and treatments with AMA and 3F2, but not cytotoxicity (Supplementary Fig. S3).

MPV stimulation promoted activation of monocyte subsets, particularly patrolling monocytes, as reflected by increased HLA-DR expression [7, 28]. Treatment with the 3F2 derivative reduced HLA-DR expression while preserving classical and proinflammatory monocyte frequencies, suggesting a selective immunomodulatory effect rather than broad immune suppression. This targeted modulation may contribute to attenuation of inflammatory responses induced by stimulation with inactivated MPV while maintaining basal monocyte homeostasis, a desirable characteristic for immunomodulatory interventions.

MPV stimulation altered the distribution of the NK cell subset, increasing CD56^Bright^CD16 + cells, although the percentage of CD56^Dim/neg^CD16^+^ populations remained unchanged. Despite their expansion, CD56^Bright^NK cells showed reduced activation, associated with reduced IL-18, consistent with impaired functional response [33, 34]. Treatment with AMA demonstrated a non-significant but potential trend towards restoring activation of the NK cell subset (*p* = 0.0625). Simultaneously, the 3F2 derivative further increased CD56^Bright^CD16^+^ frequencies and restored CD69 expression and showed a trend towards restoring HLA-DR expression (*p* = 0.0625), indicating recovery of NK cell activation and the functional profile of this population. The same profile of reduced activation of NK subsets and the proinflammatory cytokine IL18 was previously observed by Born and collaborators [33], and this profile was associated with one of the possible mechanisms by which poxviruses escape the host immune response.

NKT cells, which bridge innate and adaptive immunity, were also affected by MPV stimulation, showing reduced activation and downregulation of early (CD69) and late (HLA-DR) activation markers. Treatment with the 3F2 derivative partially restored CD69 and HLA-DR expression, although without statistical significance, suggesting a modest but selective immunomodulatory effect. Collectively, these findings suggest that 3F2 selectively counteracts MPV-induced dysregulation in immune populations, including NK and NKT cells, without inducing excessive immune activation.

Consistent with previous reports, MPV stimulation induces a dysregulated immune response characterized by broad suppression of antiviral and pro-inflammatory mediators, reflecting a classic profile of immune evasion of orthopoxvirus infections [34–36]. In our model, MPV markedly reduced the secretion of key Th1-associated cytokines, regulatory mediators, and growth factors, likely impairing effector cell function, particularly NK cell activation and cytokine production, consequently favoring viral persistence [37, 38].

Importantly, treatment with the 3F2 derivative selectively restored multiple mediators suppressed by MPV, including CXCL8 (*p* = 0.0625), IFN-γ (*p* = 0.0625), IL-12(p40), IL-15, VEGF, and NGF-β. In our model, MPV stimulation reduced CXCL8, IFN-γ, and IL-15 levels, consistent with orthopoxvirus immune evasion strategies that dampen innate cell recruitment and impair NK cell survival and antiviral function [34, 38, 39]. While clinical studies of mpox patients have not consistently shown suppression of CXCL8 upon infection, our data demonstrate that MPV stimulation in our experimental system significantly reduced CXCL8 levels compared to mock [38]. The coordinated recovery of these mediators suggests that 3F2 promotes reactivation of innate antiviral immunity without inducing excessive inflammation.

MPV did not uniformly reduce all immune mediators. CCL2 levels increased after MPV stimulation, consistent with its previously reported role in monocyte recruitment during viral infections [38, 40, 41]. Treatment with 3F2 further increased CCL2 levels, suggesting amplification of monocyte recruitment signals. Importantly, although CCL2 may contribute to early antiviral immune responses, excessive CCL2 activation has also been associated with excessive macrophage infiltration and inflammatory tissue damage during viral infections, particularly respiratory infections [42, 43]. Further studies with the kinetics of CCL2 production upon MPV stimuli are yet required to fully understand the role of this CC-chemokine in mpox infection and treatment.

Interestingly, CXCL10, which increased after MPV stimulation, was attenuated by AMA treatment but not by 3F2, indicating a specific immunomodulatory profile of AMA that may limit excessive immune activation. This CXCL10 profile has previously been observed in acute mpox patients [38].

Moreover, other immune mediators, including IL-2, IL-9, IL-10, IL-16, IL-18, TNF-α, CCL4, CCL11, and CXCL12, showed profiles of modulation after treatment with 3F2, although they did not display statistical significance, supporting a broader reorganization of the immune system.

In fact, the immune responses induced by inactivated MPV may involve the recognition of viral PAMPs by pattern recognition receptors, including Toll-like receptors (TLRs) and the cGAS-STING pathway, followed by the activation of NF-κB, MAPK, and JAK/STAT signaling pathways [5, 7]. Previous studies have demonstrated that inactivated vaccinia virus can trigger early signaling associated with MEK/ERK and c-Fos expression, suggesting that viral structural components can activate host signaling independently of productive viral replication [44, 45]. Furthermore, given that 3F2 is an aminoadamantane derivative related to amantadine, a known NMDA receptor inhibitor, the potential interaction between NMDA-associated signaling and the MAPK pathway may also contribute to its immunomodulatory effects [46].

Together, these data reveal that stimulation of PBMCs with inactivated MPV induces an altered immune profile characterized by impaired activation patterns and reduced production of key antiviral mediators, whereas treatment with 3F2 partially reverses these effects by restoring immune mediator production, modulating immune cell activation, and preserving immune balance. This finely tuned immunomodulatory profile, combined with its previously described antiviral activity against MPV [23], reinforces the potential of 3F2 as a dual-action therapeutic candidate. While current antivirals against MPV focus on limiting viral replication through direct antiviral mechanisms, different host-directed and immunomodulatory approaches have been proposed, including ribavirin, pidotimod, thymosin, IFN-β, and IL-15, although their therapeutic potential against MPV remains to be characterized in more detail [47, 48]. In this context, the ability of 3F2 to combine antiviral activity with the modulation of multiple innate immune populations and mediators highlights its potential as a complementary host-directed therapeutic approach for MPV.

This study has some limitations. The use of chemically and physically inactivated MPV does not fully replicate the complexity of infection induced by replication-competent virus, which may influence host immune recognition and the secretion of immune mediators.

Furthermore, the virus preparation was obtained from clarified viral stocks without additional purification, and the potential contribution of residual host-cell components to the observed immune responses cannot be completely excluded. However, as virus-stimulated experimental groups received the same viral preparation, the comparisons among treatment groups remain valid within the experimental framework used in this study. Thus, the immunomodulatory effects described here should be interpreted within the context of the experimental model employed. Although the use of live MPV would provide a more comprehensive assessment of host–virus interactions, experiments with replication-competent virus require Biosafety Level 3 (BSL-3) containment facilities, which may limit the feasibility of such studies. In addition, the relatively small number of healthy donors may limit the generalizability of the findings, given the interindividual variability of human immune responses.

Despite these limitations, our findings support further investigation of the immunomodulatory effects of 3F2 in the context of MPV infection. Future studies using larger cohorts, in vivo models, and replication-competent MPV will be important to validate and expand upon these observations. In this regard, we are currently developing an oral formulation of 3F2 and evaluating its stability, in vivo tolerability, and antiviral activity in live MPV infection assays performed in a BSL-3 laboratory.

## Conclusion

The findings of this work broaden the understanding of the 3F2 derivative of AMA beyond its previously reported antiviral activity against MPV. In addition to impairing virion assembly, 3F2 restores the immune response disrupted by MPV stimulation by modulating key immune cell populations (monocytes, NK cells, and NKT cells) and rebalancing immune soluble mediators involved in antiviral defense. This dual mode of action positions 3F2 as a promising therapeutic candidate for MPV infection, with potential to simultaneously affect viral replication and reverse virus-induced immune dysregulation.

## Supplementary Information

Below is the link to the electronic supplementary material.

Supplementary Material 1

## Data Availability

No datasets were generated or analysed during the current study.
