# Supplementary material for: The aminoadamantane derivative 3F2 modulates monocyte and NK cell activation and restores immune mediators altered by Monkeypox virus stimuli
Source: Inflamm Res. 2026 Oct 17;75(1):244. doi: 10.1007/s00011-026-02387-3 (PMC13676534; doi:10.1007/s00011-026-02387-3)
Supplement: Supplementary file 1 [file 11_2026_2387_MOESM1_ESM.docx]

**The aminoadamantane derivative 3F2 modulates monocyte and NK cell activation and restores immune mediators altered by Monkeypox virus stimuli**

Thaís de Fátima Silva Moraes^1,2^, Geovane Marques-Ferreira^1,2^, Ágata Lopes-Ribeiro^1,2^, Erik Vinicius de Sousa Reis^1,2^, Gabriela Lorrany Aparecida Azevedo^1,2^, Victor de Melo Rocha^1,2^, Marília Bueno da Silva Menegatto^1,2^, Felipe Alves Clarindo^1,2^, Caio Wilker-Teixeira^1,2^, Laura Cardoso Corrêa-Dias^1,2^, Nathália Evelyn Morais Costa^3^, Luan Ramalho Pinheiro^3^, Adriano de Paula Sabino^4^, Erna Geessien Kroon^1^, Ângelo de Fátima^3^, Jordana Grazziela Alves Coelho-dos-Reis^1,2^*

^1^ PROSPERA Group, Grupo de Prospecção e Pesquisas em Resposta Imune e Antivirais, Universidade Federal de Minas Gerais, Belo Horizonte, MG.

^2^ Departamento de Microbiologia, Instituto de Ciências Biológicas, Universidade Federal de Minas Gerais, Belo Horizonte, MG.

^3^ Departamento de Química, Instituto de Ciências Exatas, Universidade Federal de Minas Gerais, Belo Horizonte, MG.

^4^ Laboratório de Hematologia Clínica, Experimental e Molecular, Departamento de Análises Clínicas e Toxicológicas, Faculdade de Farmácia, Universidade Federal de Minas Gerais, Belo Horizonte, MG.

* Corresponding Author: Jordana Grazziela Alves Coelho-dos-Reis

E-mail address: [reisjordana@gmail.com](mailto:reisjordana@gmail.com)

Laboratório de Virologia Básica e Aplicada, Departamento de Microbiologia, Instituto de Ciências Biológicas, Universidade Federal de Minas Gerais, Avenida Antônio Carlos, 6627 – Pampulha – Belo Horizonte – Minas Gerais – 31270-901, Brazil. Tel.: + 55 31 3409 2745.

**SUPPLEMENTARY MATERIAL**

**Table S1.** Surface staining antibody mix

| **Antibody-Fluorochrome and (Clone)** |  | **Brand** | **Cat** | **Dilution** |
| --- | --- | --- | --- | --- |
| anti-CD3–PE-Cy7 (SK7) |  | BD | #557851 | 1:50 |
| anti-CD4–PE (EDU-2) |  | Immunotools | #21278044 | 1:50 |
| anti-CD14–APC (MEM-15) |  | Immunotools | #21620146 | 1:50 |
| anti-CD16–AF 700 (3G8) |  | BD | #557920 | 1:50 |
| anti-CD56–V450 (B159) |  | BD | #560360 | 1:50 |
| anti-CD69–APC-H7 (FN50) |  | BD | #557756 | 1:50 |
| anti-HLA-DR–PerCP (L243) |  | BD | #339205 | 1:10 |
| anti-CD19–FITC (HIB19) |  | BD | #555412 | 1:50 |

**Table S2.** Interquartile ranges (25th–75th percentiles) for the AMA and 3F2 control groups.

|  |  | |  | | Mock | | AMA | | 3F2 | |
| --- | --- | --- | --- | --- | --- | --- | --- | --- | --- | --- |
| Monocyte | Classic | | % | | 71.13 – 79.15 | | 39.1 – 51.5 | | 37.8 – 53.4 | |
|  |  | | HLA-DR (gMFI) | | 10856 - 23779 | | 22766 – 30949 | | 22076 – 28236 | |
|  |  | | % HLA-DR^+^ | | 89.90 - 98.58 | | 96.55 – 99.45 | | 97.13 – 98.60 | |
|  |  | | % HLA-DR^High^ | | 3.10 – 13.35 | | 35.45 – 54.55 | | 32.85 – 44.45 | |
|  | Proinflammatory | | % | | 17.85 – 23.10 | | 45.20 – 73.50 | | 51.15 – 71.93 | |
|  |  | | HLA-DR (gMFI) | | 11401 – 25900 | | 44457 – 59279 | | 48938 – 65865 | |
|  |  | | % HLA-DR^+^ | | 93.95 – 98.80 | | 99.35 – 99.75 | | 99.30 – 99.83 | |
|  |  | | % HLA-DR^High^ | | 8.005 – 23.68 | | 59.20 – 70.80 | | 52.05 – 69.10 | |
|  | Patrolling | | % | | 0.89 – 2.09 | | 1.95 – 5.22 | | 1.48 – 3.35 | |
|  |  | | HLA-DR (gMFI) | | 13728 – 19483 | | 10819 – 18764 | | 11572 – 20205 | |
|  |  | | % HLA-DR^+^ | | 75.53 – 90.43 | | 67.03 – 83.63 | | 66.48 – 87 | |
|  |  | | % HLA-DR^High^ | | 20.83 – 28.43 | | 40.38 – 48.18 | | 37.98 – 46.28 | |
| NK | CD56^Bright^NK | | % | | 1.82 – 4.46 | | 4.34 – 6.55 | | 3.24 – 5.813 | |
|  |  | | % CD69^+^ | | 50.38 – 52.90 | | 55.95 – 66.80 | | 47.25 – 65.65 | |
|  |  |  | CD69 gMFI | | 49.65 – 81.90 | | 76.08 – 109.8 | | 65.4 – 115.8 | |
|  |  |  | % HLA-DR^+^ | | 41.78 – 53 | | 43.33 – 59.4 | | 49.35 – 59.98 | |
|  |  |  | HLA-DR gMFI | | 1247 - 1680 | | 1518 – 2262 | | 1623 – 2168 | |
|  | CD56^Bright^CD16^+^NK | | % | | 1.89 – 5.83 | | 39.88 – 74.50 | | 37.78 – 66.33 | |
|  |  | | % CD69^+^ | | 23.80 – 50 | | 47.8 – 66.55 | | 36.78 – 66.13 | |
|  |  |  | CD69 gMFI | | 58.20 – 333 | | 80.03 – 100.1 | | 64.08 – 119 | |
|  |  |  | % HLA-DR^+^ | | 46.60 – 78.55 | | 43.28 – 58.85 | | 43.75 – 60.83 | |
|  |  |  | HLA-DR gMFI | | 1123 - 3188 | | 1405 - 2096 | | 1569 – 2023 | |
|  | CD56^Dim^NK | | % | | 25.48 – 50.83 | | 23.68 – 56.18 | | 15.38 – 53.85 | |
|  |  | | % CD69^+^ | | 37.08 – 47.25 | | 35.1 – 46.48 | | 34.53 – 40.7 | |
|  |  |  | CD69 gMFI | | 52.88 – 254.3 | | 112 – 130.3 | | 98.7 – 141.5 | |
|  |  |  | % HLA-DR^+^ | | 21.80 – 38.53 | | 28.65 – 39.98 | | 30.08 – 40.3 | |
|  |  |  | HLA-DR gMFI | | 855.5 – 1343 | | 1143 - 1515 | | 1206 – 1471 | |
|  | CD56^Dim^CD16^+^NK | | % | | 5.43 – 14.88 | | 95.75 – 98.45 | | 95.73 – 97.98 | |
|  |  | | % CD69^+^ | | 69.38 – 90.05 | | 64.55 – 75.83 | | 62.63 – 72.28 | |
|  |  |  | CD69 gMFI | | 209 – 534.5 | | 110.5 – 129.8 | | 97.33 – 141.5 | |
|  |  |  | % HLA-DR^+^ | | 31 – 56.70 | | 29.23 – 39.75 | | 31.83 – 40.15 | |
|  |  |  | HLA-DR gMFI | | 1160 – 2293 | | 1142 – 1497 | | 1171 – 1466 | |
|  | CD56^Neg^NK | | % | | 43.95 – 66.78 | | 36.43 – 68.15 | | 37.85 – 78.45 | |
|  |  | | % CD69^+^ | | 61.25 – 72.20 | | 72.43 – 85.55 | | 74.53 – 88.30 | |
|  |  |  | CD69 gMFI | | 47.60 – 114.50 | | 232 – 306.5 | | 221 – 348 | |
|  |  |  | % HLA-DR^+^ | | 55.40 – 70.28 | | 37.23 – 55.68 | | 30.90 – 48.93 | |
|  |  |  | HLA-DR gMFI | | 9315 – 10688 | | 3420 – 4983 | | 2806 – 8495 | |
|  | CD56^Neg^CD16^+^NK | | % | | 5.138 – 11.45 | | 20.3 – 30.9 | | 18.33 – 33.25 | |
|  |  | | % CD69^+^ | | 48.23 – 74.20 | | 59.28 – 74.03 | | 58.43 – 78.05 | |
|  |  | | CD69 gMFI | | 118.8 – 267.5 | | 165.3 – 217.5 | | 161.5 – 250.8 | |
|  |  |  | % HLA-DR^+^ | | 85.05 – 93.43 | | 59.8 – 74.03 | | 58.43 – 78.05 | |
|  |  |  | HLA-DR gMFI | | 15229 - 26805 | | 2429 - 6997 | | 2257 - 5518 | |
| NKT |  | | % | | 0.08 – 0.165 | | 0.075 – 0.157 | | 0.077 – 0.218 | |
|  | | | % CD69^+^ | | 84.80 – 92.68 | | 52.38 – 79.48 | | 43.03 – 72.48 | |
|  |  |  | CD69 gMFI | | 239.8 – 663 | | 140 – 347 | | 116.4 – 239.8 | |
|  |  |  | % HLA-DR^+^ | | 72.50 – 87.73 | | 44.3 – 58.7 | | 49.58 – 54.58 | |
|  |  |  | HLA-DR gMFI | | 1413 - 4526 | | 1545 – 2720 | | 1544 – 2127 | |
| Lymphocyte | |  | % | | 40.10 – 67.50 | | 21.50 – 47.43 | | 23.13 – 47.60 | |
|  | CD3^-^ | | % | | 16.5 – 30.85 | | 15.7 – 26.95 | | 16.15 – 29.85 | |
|  | LB CD3^-^ CD19^+^ | | % | | 21.97 – 40.53 | | 5.47 – 12.4 | | 4.84 – 9.83 | |
|  |  | | % CD69^+^ | | 53.35 – 72.38 | | 67.55 – 71.53 | | 67.05 – 71.85 | |
|  |  | | CD69 gMFI | | 56.63 – 147.3 | | 122.5 – 139.3 | | 107.5 – 131.3 | |
|  |  | | % HLA-DR^+^ | | 83.65 – 96.43 | | 96.65 – 98.68 | | 96.20 – 99.18 | |
|  |  | | HLA-DR gMFI | | 15428 – 26487 | | 58899 – 71285 | | 52293 – 73575 | |
|  | CD3^+^ | | % | | 68.18 – 82.73 | | 71.95 – 83.30 | | 69.08 – 84.93 | |
|  | LTh CD3^+^ CD4^+^ | | % | | 56.40 – 63.05 | | 54.88 – 65.50 | | 55.10 – 66.20 | |
|  |  | | % CD69^+^ | | 52.58 – 57.33 | | 55.08 – 63.60 | | 54.35 – 64.35 | |
|  |  | | CD69 gMFI | | 62 – 83.35 | | 82.45 – 103.3 | | 75.88 – 108.3 | |
|  |  | | % HLA-DR^+^ | | 3.53 – 5.68 | | 5.84 – 8.14 | | 4.99 – 8.39 | |
|  |  | | HLA-DR gMFI | | 1444 – 1727 | | 1780 – 2118 | | 1621 - 2083 | |
|  | LTctx CD3^+^ CD8^+^ | | % | | 37.75 – 52 | | 34.38 – 44.98 | | 33.33 – 44.73 | |
|  |  | | % CD69^+^ | | 55.45 – 63.48 | | 64.60 – 68.70 | | 63.40 – 69.25 | |
|  |  | | CD69 gMFI | | 48.45 – 72.45 | | 107.5 – 111.5 | | 96.25 – 118 | |
|  |  | | % HLA-DR^+^ | | 3.06 – 6.45 | | 9.71 – 13.40 | | 8.63 – 12.70 | |
|  |  | | HLA-DR gMFI | | 1109 - 1526 | | 1848 – 2191 | | 1704 – 2137 | |
| Soluble immune mediators | Chemokines  (pg/mL) | | CCL2 | | 3.11 – 5.16 | | 2.47 – 12.21 | | 4.82 – 10.89 | |
|  |  |  | CXCL8 | | 3.16 – 10.88 | | 103.7 – 351.8 | | 157.9 – 706.4 | |
|  |  |  | CXCL10 | | 8.78 – 13.63 | | 22.16 – 61.0 | | 27.23 – 49.17 | |
|  |  |  | CCL4 | | 69.25 – 86.20 | | 88.24 – 144.9 | | 68.66 – 158.5 | |
|  |  |  | CCL11 | | 1.60 – 2.7 | | 1.75 – 3.08 | | 1.33 – 2.38 | |
|  |  |  | CXCL12 | | 88.09 – 112 | | 63.94 – 98.96 | | 70.08 – 81.79 | |
|  | Proinflammatory cytokines  (pg/mL) | | IL-15 | | 462.4 – 512.3 | | 497.3 – 530.7 | | 466.5 – 525.3 | |
|  |  |  | IL-12-p40 | | 13.93 – 23.08 | | 18.92 – 29.03 | | 16.53 – 29.14 | |
|  |  |  | IFN-γ | | 7.48 – 8.05 | | 10.58 – 46.22 | | 19.41 – 33.24 | |
|  |  |  | TNF-α | | 20.51 – 24.70 | | 14.87 – 31.73 | | 24.70 – 28.51 | |
|  |  |  | IL-16 | | 91.97 – 108-.6 | | 57.31 – 174.5 | | 53.92 – 158.9 | |
|  | Regulatory cytokines  (pg/mL) | | IL-5 | | 86.48 – 106.1 | | 113.3 – 134.0 | | 89.86 – 99.05 | |
|  |  |  | IL-18 | 2.99 – 3.78 | | 1.78 – 2.94 | | 1.58 – 2.63 | |  |
|  |  |  | IL-9 | | 87-02 – 115.5 | | 37.92 – 52.99 | | 36.07 – 56.07 | |
|  |  |  | IL-10 | | 2.12 – 2.50 | | 6.83 – 8.79 | | 5.81 – 9.17 | |
|  | Growth factors  (pg/mL) | | VEGF | | 70.32 – 156.5 | | 150.9 – 293.2 | | 100.1 – 248.5 | |
|  |  |  | β-NGF | | 0.12 – 0.24 | | 0.09 – 0.34 | | 0.23 – 0.29 | |
|  |  |  | IL-2 | | 1.48 – 1.75 | | 1.85 – 6.36 | | 2.26 – 3.07 | |

**Figure S1. Treatment with AMA or 3F2 did not reverse MPV-induced suppression of early and late activation profiles in CD56^Dim/neg^NK cell subsets.** Activation profile of a) CD56^Dim^CD16^+^NK and b) CD56^Neg^CD16^+^NK subsets based on CD69 and HLA-DR expression, shown as frequency (percentage) and geometric mean fluorescence intensity (gMFI). Data obtained from six independent donors (n = 6) are presented in a box with minimum to maximum values. Statistical significance was determined using the Wilcoxon test, with * *p* < 0.05 indicating significant differences.

**Figure S2. AMA and 3F2 did not restore lymphocyte activation after MPV stimulation** a) Flow cytometry gating strategy for identification of total lymphocytes, B cells (CD3⁻CD19⁺), and T cell subsets (CD4⁺, CD8⁺), with evaluation of CD69 and HLA-DR expression. b) Frequency of total lymphocytes, B cells, CD4⁺T cells, and CD8⁺T cells across conditions. Frequency and activation profile of lymphocyte subsets c) LB CD3⁻CD19⁺, d) LTh CD3⁺CD4⁺, and e) LTctx, CD3⁺CD8⁺ based on CD69 and HLA-DR expression, shown as frequency (percentage) and geometric mean fluorescence intensity (gMFI). Data obtained from six independent donors (n = 6) are presented in a box with minimum to maximum values. Statistical significance was determined using the Wilcoxon test, with * *p* < 0.05 indicating significant differences.

**Figure S3.** Cell viability of PBMCs following stimulation with MPV and treatment with AMA or 3F2. **a)** Representative plots showing the cell viability of PBMCs cultured under Mock, MPV, MPV + AMA, and MPV + 3F2 conditions after 12h of stimuli. The percentage of viable cells is indicated within each gate. **b)** Overlay histograms of live staining and **c)** a viability bar graph is presented. No substantial differences in cell viability were observed among the experimental conditions, indicating that MPV stimuli and treatment with AMA or 3F2 did not induce detectable cytotoxic effects under the conditions evaluated.
